# Synergistic Co-Blending of Low- and High-Rank Coals: A Strategy for Engineering High-Performance Porous Carbons for Supercapacitors

**DOI:** 10.3390/molecules31172997

**Published:** 2026-08-27

**Authors:** Yucong Yun, Rui Sheng, Ting Liu

**Affiliations:** State Key Laboratory of Chemistry and Utilization of Carbon Based Energy Resources, College of Chemistry, Xinjiang University, Urumqi 830017, China; huaxueyyc@163.com

**Keywords:** supercapacitors, coal blending strategy, hierarchical porous carbon

## Abstract

Owing to the inherent limitations of a single coalification degree, precursors derived from a single-rank coal often struggle to simultaneously fulfill the requirements of composition, structure, and performance. To address this, a blended-coal strategy combining oxygen-rich low-rank Baishihu coal and aromatic-rich high-rank Yicheng coal is proposed to overcome the reactivity–stability trade-off during KOH/KCl activation. The optimized sample, BYCM-40, delivers a high specific surface area of 2556.53 m^2^·g^−1^, a hierarchical porosity with 75% micropores and 25% mesopores, and 22.77 at% surface oxygen, achieving a specific capacitance of 357.8 F·g^−1^ and remarkable cycling stability with 99.2% retention after 20,000 cycles. This work is expected to open a sustainable pathway for valorizing low-rank coals into high-performance porous carbons for advanced supercapacitors.

## 1. Introduction

Increasing needs for high-efficiency energy storage have driven widespread investigation into next-generation devices. Among the various options, supercapacitors are particularly attractive because of their extended operational lifespan, superior power density, and rapid recharge capability, which has led to their broad adoption in hybrid electric vehicles and portable electronics. According to their charge-storage principles, supercapacitors are typically divided into two classes: electrical double-layer capacitors (EDLCs) and pseudocapacitors (PCs) [1]. In EDLCs, energy is stored through the swift adsorption and desorption of ions occurring at the boundary between the electrolyte and the electrode [2]. A strong relationship exists between the specific capacitance of electrode materials and their specific surface area (SSA) as well as their pore architecture. Since the electrode material largely dictates the performance of supercapacitors, current research efforts are concentrating on the optimization of preparation routes and the identification of appropriate carbon precursors for the fabrication of high-performance porous carbon electrodes. Consequently, porous carbon featuring a high SSA is extensively utilized for EDLC electrodes because of its low cost, chemical robustness, and favorable electronic conductivity [3,4].

In contrast to EDLC-type porous carbon electrodes that store charge through physical ion adsorption/desorption, battery-type and hybrid energy storage systems rely on Faradaic reactions to pursue higher energy densities, making electrode/electrolyte interfacial engineering a critical common challenge. In the field of aqueous zinc-ion batteries, Li and Han demonstrated that lithium nitrate (LiNO_3_) as a multifunctional additive can regulate Zn^2+^ solvation and promote controlled deposition, achieving over 500 stable cycles with an average Coulombic efficiency of 99.7% [5]. A comprehensive review by Zhao et al. systematically summarized four cathode energy storage mechanisms (Zn^2+^ insertion/extraction, H^+^/Zn^2+^ co-intercalation, chemical conversion, and dissolution/deposition) along with anode optimization strategies including artificial protective layers, electrolyte additives, and 3D porous structures [6]. In the domain of solid-state lithium batteries, Chen et al. reviewed the performance enhancement mechanisms of metal–organic framework (MOF)–polymer composite electrolytes, highlighting interface engineering strategies such as in situ polymerization, in situ MOF growth, gel-like ionic conductors, and composite cathode design to improve interfacial compatibility and stability [7]. These studies underscore that interfacial engineering is a ubiquitous challenge across diverse battery chemistries. Compared with these Faradaic-reaction-dependent systems, EDLC-type porous carbon electrodes offer inherent advantages in fast response and ultra-long cycle life, though their energy density remains relatively low. In this context, the present study aims to enhance the charge-storage capability of carbon-based EDLC electrodes through pore structure engineering of coal-derived hierarchically porous carbons, offering a new design strategy for high-performance, long-life electrode materials.

The electrochemical performance of the resultant porous carbon is jointly governed by both the composition of the carbon precursors and the techniques employed for their preparation. As a non-uniform carbon source possessing an intricate chemical architecture, coal consists predominantly of polycyclic aromatic hydrocarbons (PAHs), to which aliphatic chains and ether linkages are attached at their edges. This material provides benefits including economical affordability and a substantial carbon fraction [8]. According to their volatile matter level and rank of coalification, coals are customarily categorized into anthracite (high rank), bituminous coal (medium rank), and lignite (low rank). Within this classification, low-rank coals typically possess a greater abundance of oxygen-bearing functional groups and a larger proportion of volatile material. In contrast, high-rank coals show an elevated coalification degree, a higher carbon fraction, and a reduced specific surface area. However, these distinct characteristics impose inherent limitations when a single coal rank is employed as the sole precursor. The oxygen-rich functionalities in low-rank coals can induce substantial pseudocapacitance and improve electrolyte wettability, yet their excessive volatile content often leads to severe foaming, low carbon yield, and underdeveloped porosity during activation. Conversely, the condensed aromatic frameworks of high-rank coals provide structural stability and facilitate the formation of high specific surface areas, but their scarcity of heteroatoms limits Faradaic contributions and results in poor surface wettability. To overcome this reactivity–stability trade-off and synergistically combine the advantages of both ranks, a blended-coal strategy is developed, using oxygen-rich low-rank Baishihu coal and aromatic-rich high-rank Yicheng coal. This strategy aims to couple the pseudocapacitive oxygen functional groups and favorable wettability of low-rank coal with the robust carbon skeleton and pore-forming capability of high-rank coal, thereby enabling the synthesis of porous carbons with balanced pore structure, high specific surface area, and abundant electroactive sites for advanced supercapacitor electrodes.

At present, the principal approaches for synthesizing coal-derived porous carbon comprise physical activation, chemical activation, and template-based techniques. The choice of coal type significantly influences both the chemical composition and the capacitive behavior of the resultant porous carbon. Employing a mixture of sodium chloride and zinc chloride as both a template and an activating agent, Chen and co-workers carbonized Kuche bituminous coal sourced from Xinjiang under a nitrogen flow [9]. Zinc chloride at elevated temperatures created micropores across the carbon skeleton’s surface, whereas sodium chloride served as a mesopore-directing template. The resulting porous carbon material exhibited a yield of 42.9 wt% along with a specific capacitance of 344.3 F·g^−1^. Ren and colleagues adopted low-rank lignite as a carbon precursor and synthesized hierarchically porous carbon through KOH-assisted activation [10]. At elevated temperatures, the etching action of KOH on the carbon precursor produces micropores. A supercapacitor constructed using this material delivered a power density of 999.8 W·kg^−1^ when tested at 1 A·g^−1^, along with an energy density of 19.4 Wh·kg^−1^. Following 10,000 charge–discharge cycles performed at a current density of 2 A·g^−1^, the Coulombic efficiency of the device stayed near 100%. Despite these advances, a single-rank coal precursor inherently struggles to reconcile the conflicting demands of structural stability and sufficient electroactivity—low-rank coals possess abundant functional groups yet tend to collapse during activation, whereas high-rank coals form robust porous frameworks but lack surface functionality. This limitation makes it difficult to simultaneously optimize pore architecture, surface chemistry, and capacitive performance.

Blending high- and low-rank coals offers compelling advantages in resolving these limitations: it reduces raw-material cost, increases carbon yield, and enables precise control over pore architecture. Blending coals of different ranks allows the pore size distribution to be tuned by simply adjusting the blend ratio, as demonstrated in prior studies [11,12,13]. In the present case, low-rank Baishihu coal, with its high volatile matter, abundant aliphatic groups, and plentiful oxygen-containing functionalities [14,15,16], supplies reactive sites for the activator and promotes extensive pore formation. In contrast, high-rank YCM coal, characterized by an advanced coalification degree and abundant polycyclic aromatic hydrocarbons (PAHs), stabilizes the carbon framework through π–π stacking interactions, enhances carbon yield [17], and suppresses excessive etching and pore collapse [18]. The synergistic cooperation between the two coals generates a hierarchically porous architecture that complements the oxygen-rich low-rank component, ultimately achieving a favorable compromise among carbon yield, pore structure, and electrochemical performance. However, there is still a lack of systematic understanding of how the blend ratio of high- and low-rank coals influences the interplay among pore structure, surface chemistry, and capacitive behavior under KOH/KCl activation. To address this gap, this study systematically varies the blend ratio to establish structure–performance correlations and to identify the optimal composition for advanced supercapacitor electrodes.

In this study, Baishihu coal and Yicheng coal were mixed at specific ratios and carbonized under a nitrogen atmosphere to prepare coal-based porous carbon via KOH/KCl activation. By tuning the blend ratio of coals possessing varying coalification degrees, porous carbon materials with superior electrochemical performance are obtainable. The present work clarifies how coal type and mixing proportion influence the chemical composition, porous architecture, and capacitive properties of the resulting products. Moreover, the evolution process of the porous carbon was explored, uncovering the underlying activation mechanism during carbonization.

## 2. Results and Discussion

### 2.1. Characterization of Raw Coals

**Figure 1:**
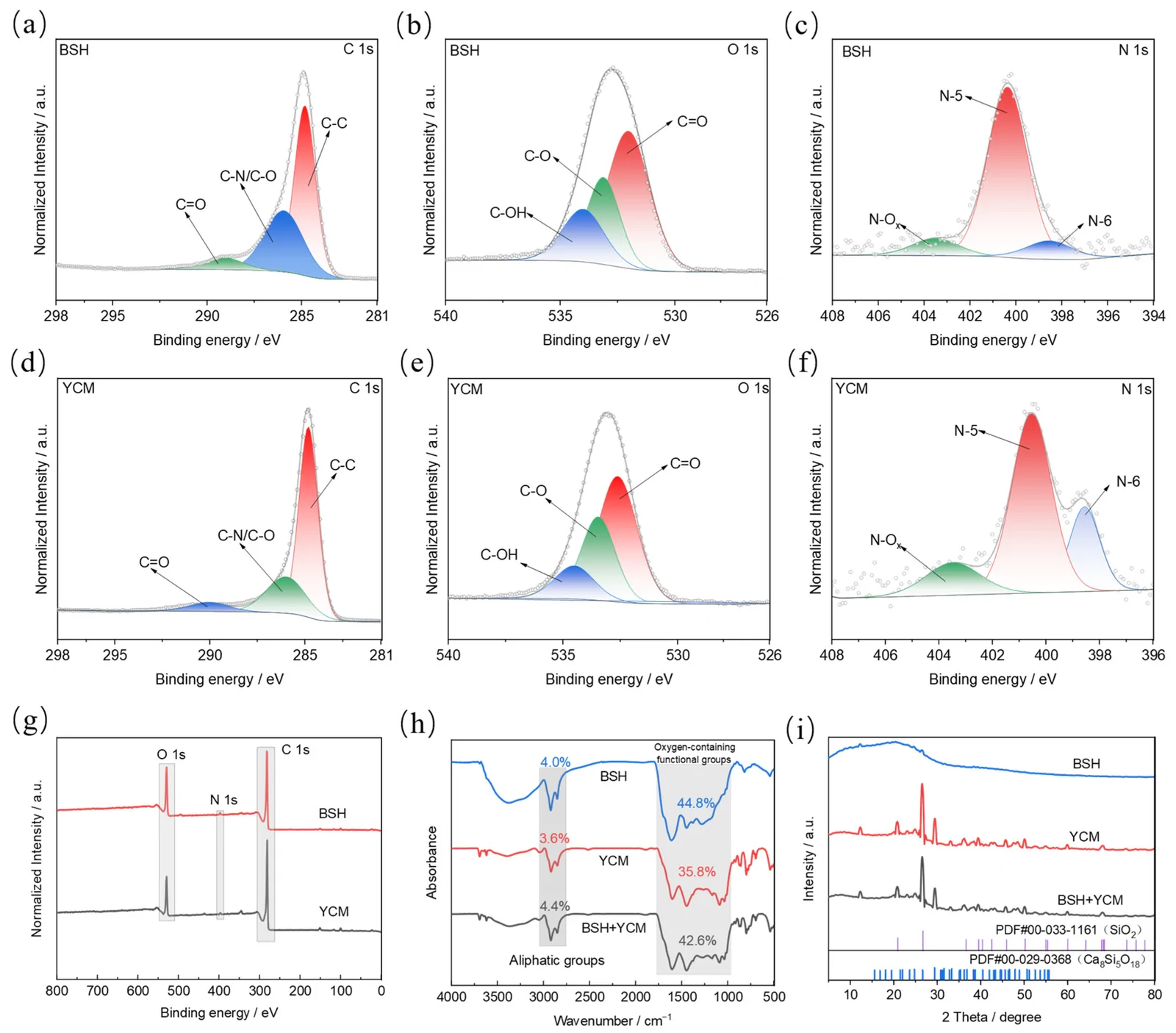
High-resolution XPS spectra of Baishihu coal: (**a**) C1s, (**b**) O1s, and (**c**) N1s. High-resolution XPS spectra of Yicheng coal: (**d**) C1s, (**e**) O1s, and (**f**) N1s. (**g**) Total XPS spectra of Baishihu coal and Yicheng coal. (**h**) IR spectra of Baishihu and Yicheng coals. (**i**) XRD patterns of Baishihu and Yicheng coals, as well as the blended coal.

Coal’s organic fraction consists primarily of carbon, hydrogen, and oxygen (>95%), with carbon content increasing with coalification degree (lignite: 60–70%; bituminous: 74–92%; anthracite: 90–98%). The diverse alkyl side chains and polycyclic aromatic hydrocarbons (PAHs) present in coals strongly influence the yield and performance of derived carbon materials [19]. Proximate analysis (Table S1) reveals that BSH coal has a higher volatile matter content (46.34 wt%) than YCM coal (23.10 wt%), consistent with its lower-rank lignite classification. FTIR spectra (Figure 1h) show that the proportion of oxygen-containing functional groups (1000–1800 cm^−1^) in BSH reaches 44.8%, substantially higher than that of YCM (35.8%). Such functional groups provide active sites for activation agents [20]; in particular, C=O and –COOH groups promote pore generation and facilitate the development of a more extensive porous network in the carbon matrix.

XPS results (Table S4) further indicate that the oxygen content of BSH coal (19.90 at%) is higher than that of YCM coal (16.52 at%). The proportion of C=O groups in BSH coal (53.16%) also exceeds that in YCM coal (51.36%). Additionally, the FT-IR spectrum (Figure 1h) shows that the aliphatic group content in YCM coal (3.6%) is lower than that in BSH (4.0%), while the BSH+YCM blend exhibits a higher aliphatic content (4.4%). It has been reported that a lower aliphatic content in coal is more favorable for achieving higher carbon yields [21]. Furthermore, the XRD pattern (Figure 1i) reveals that the main impurities in the ash of YCM coal are silicon dioxide (SiO_2_) and calcium silicate (Ca_8_Si_5_O_18_).

Owing to its low aliphatic content, YCM coal enables higher carbon yields, whereas BSH coal, containing more oxygen-containing and aliphatic functional groups, facilitates pore formation during the preparation of porous carbon materials. When these two coals are combined, the resultant blend brings together the elevated oxygen level and plentiful aliphatic groups of BSH with the superior carbon fraction of YCM that stems from its enhanced coalification degree [22], in addition to their individual alkyl side chains and oxygen-bearing functional moieties. Following activation, this cooperative interplay ultimately gives rise to coal-based porous carbon materials featuring a highly developed pore network and a modest specific surface area.

### 2.2. Mechanism of Porous Carbon Formation

**Figure 2:**
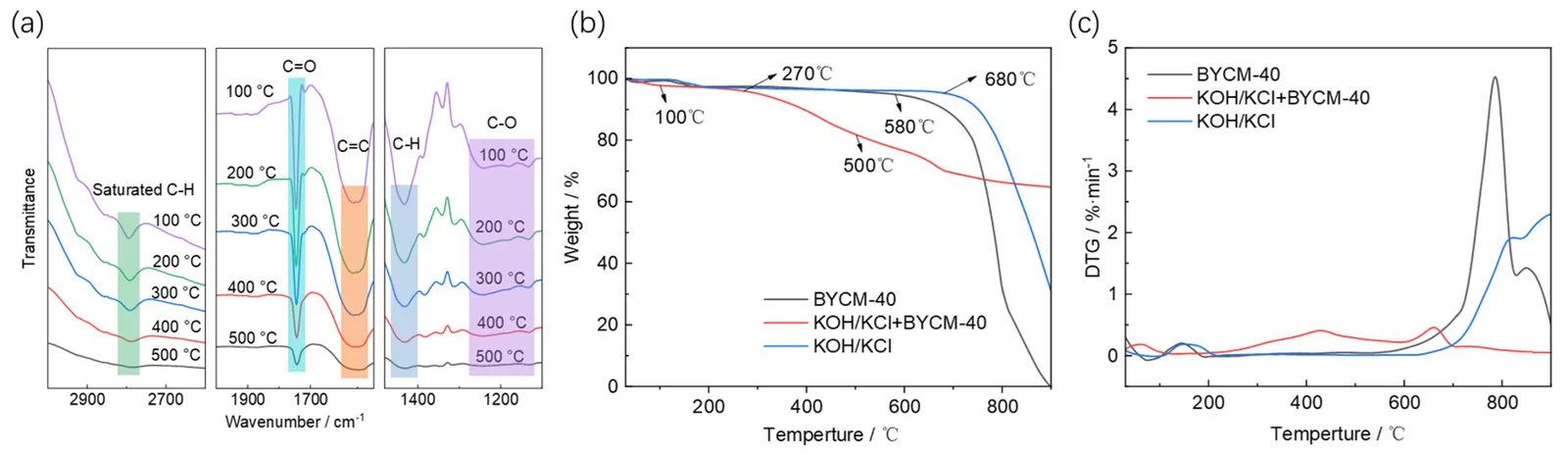
(**a**) In situ FT-IR spectra of the BYCM-40 sample with KOH/KCl during carbonization at different temperatures. (**b**) TG curves of BYCM-40, KOH/KCl+BYCM-40, and KOH/KCl. (**c**) DTG curves of BYCM-40, KOH/KCl+BYCM-40, and KOH/KCl.

In situ FTIR (Figure 2a) and thermogravimetric analysis (Figure 2b,c) elucidated the porous carbon formation mechanism. In the KOH/KCl system, KCl raised the melting point, with rapid mass loss near 680 °C, justifying 700 °C as the activation temperature. Below 200 °C, weight loss was dominated by H_2_O evolution and carboxyl decomposition [23], evidenced by diminishing –OH (∼3000 cm^−1^) and –COOH (∼1750 cm^−1^) bands. Around 270 °C, alkyl side chains detached via cleavage of aliphatic C–N/O bonds [24], confirmed by fading aliphatic –CH_x_ (∼1450 cm^−1^) and C–O (∼1200 cm^−1^) peaks (Figure 2a,b). At 600–700 °C, aromatic condensation occurred, promoted by oxygen-containing functional groups that act as crosslinking sites [25], accompanied by enhanced signals in the C=O/C=C overlap region at approximately 1600 and 1750 cm^−1^ [11]. Heat treatment subsequently drives the conversion of disordered carbon into sp^2^-hybridized graphitic microcrystallites [26].

Based on this, the influence of the coal blending ratio was examined (Table 1): higher proportions of high-rank YCM enhance PAH π–π stacking, suppress mass loss, and raise carbon yield, whereas greater additions of low-rank BSH introduce abundant oxygen functionalities and alkyl side chains that facilitate activation and pore formation, depleting the carbon framework and lowering yield. This trade-off validates the blended-coal design principle—sacrificing partial carbon yield to achieve an optimized pore structure and enhanced electrochemical performance.

### 2.3. Morphology, Composition, and Structural Characterization

**Figure 3:**
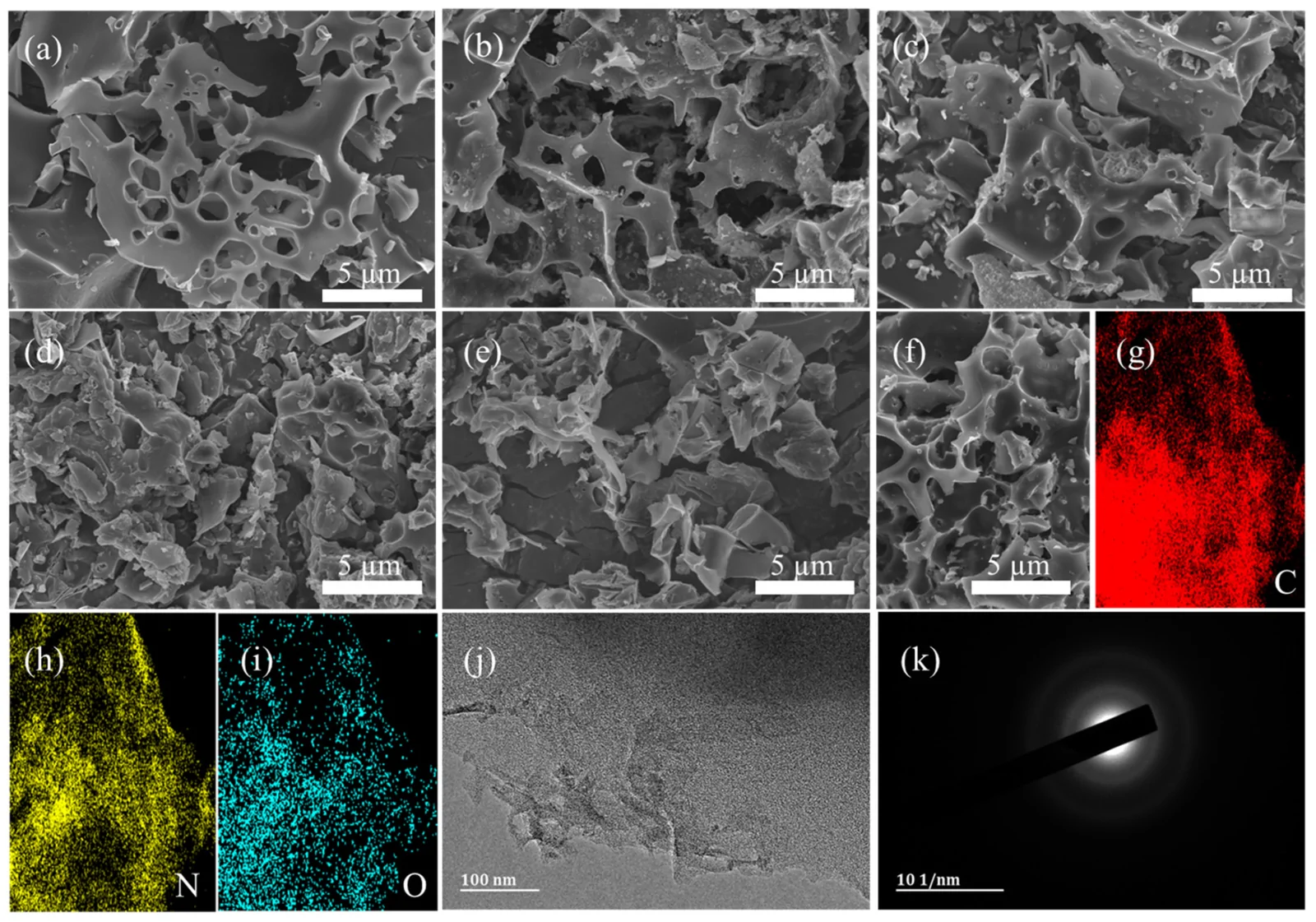
(**a**–**e**) SEM images of BSH (**a**), BYCM-20 (**b**), BYCM-40 (**c**), BYCM-60 (**d**), and YCM (**e**). (**f**–**i**) SEM image and corresponding elemental mapping images of BYCM-40. (**j**) TEM image of BYCM-40. (**k**) Electron diffraction pattern of BYCM-40.

SEM images (Figure 3) show that BSH possesses a highly porous structure, which is attributed to the abundant oxygen-bearing functional groups and alkyl side chains that supply active sites for the activator and facilitate pore formation [20]. As the YCM fraction increases, the porosity of the BYCM-X series gradually declines (Figure 3b–d). This trend arises because the growing PAH content promotes strong π–π stacking interactions, leading to the formation of ordered, compact aggregates that densify the carbon framework and hinder activator diffusion to deep reaction sites [27]. When the blending ratio is properly chosen (40 wt% BSH), BYCM-40 exhibits a comparatively abundant pore network (Figure 3c), whereas YCM (100% YCM) shows only sparse surface pores (Figure 3e). The elemental mapping images of BYCM-40 (Figure 3g–i) demonstrate that C, N, and O are homogeneously dispersed throughout BYCM-40. Transmission electron microscopy (TEM) images (Figure 3j) reveal numerous bright spots distributed within the dark gray regions of BYCM-40, corresponding to pores; no lattice fringes are observed, indicating that the material is predominantly amorphous carbon. The electron diffraction pattern (Figure 3k) features a diffuse halation without any discernible sharp diffraction spots, suggesting that the material exists in an amorphous or highly disordered state—an observation that aligns well with the XRD analysis.

**Figure 4:**
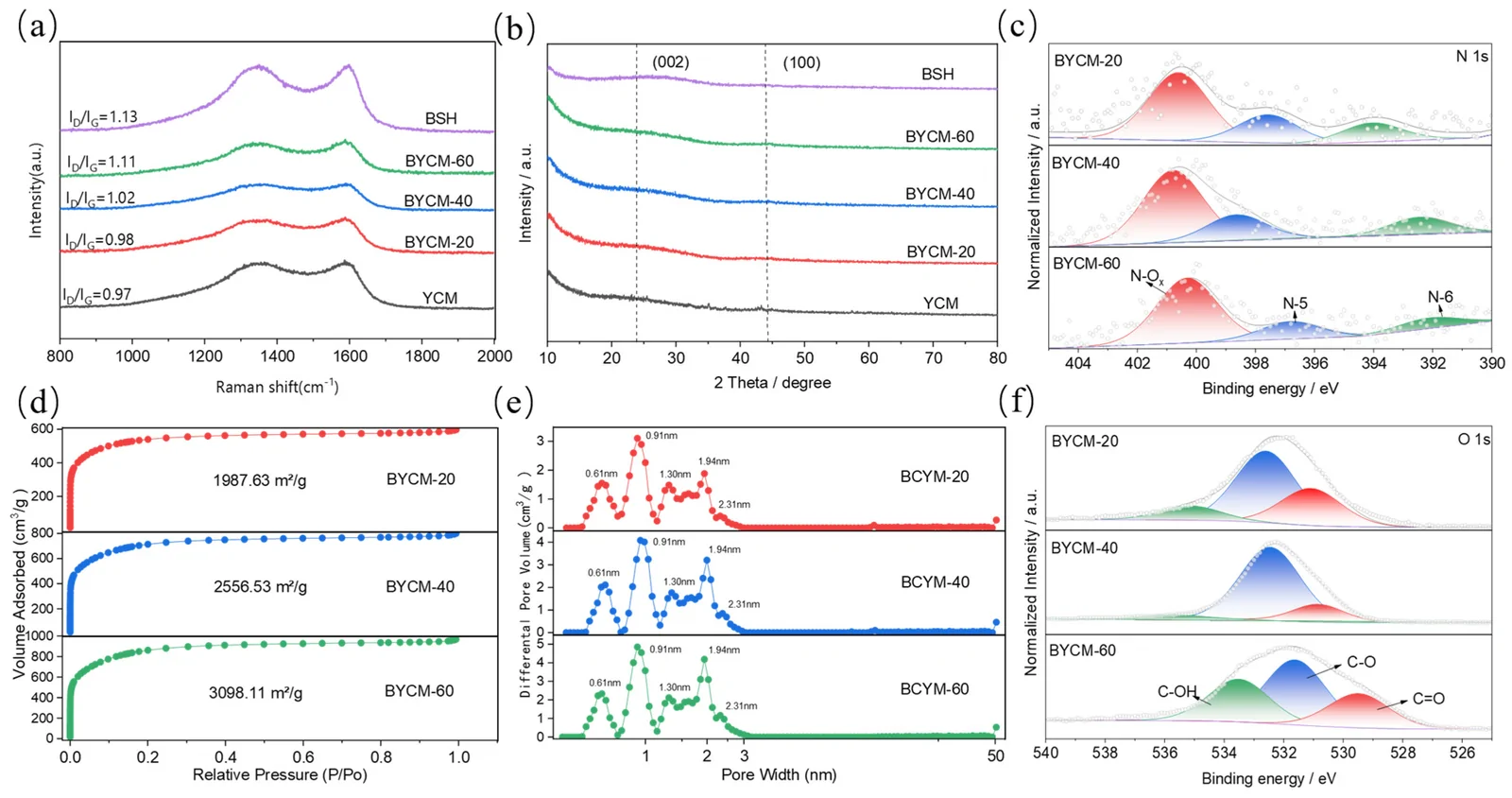
Structural characterization of BSH, BYCM-20, BYCM-40, BYCM-60, and YCM: (**a**) Raman spectra, (**b**) XRD patterns, (**c**) high-resolution N 1s XPS spectra, (**d**) N_2_ adsorption–desorption isotherms, (**e**) pore size distribution curves, and (**f**) high-resolution O 1s XPS spectra.

The X-ray diffraction (XRD) patterns presented in Figure 4b reveal two diffuse, low-intensity peaks for every sample, located at approximately 23° and 43° (2θ), which are assigned to the (002) and (100) planes of disordered carbon, respectively. These characteristics confirm that the prepared material consists primarily of disordered carbon with a low degree of graphitization [28], which is highly consistent with the electron diffraction observations (Figure 3k).

Raman spectra (Figure 4a) display D bands (~1350 cm^−1^, defect-induced sp^3^ carbon) and G bands (~1590 cm^−1^, sp^2^ graphitic carbon) [29,30]. The I_D_/I_G_ ratio decreases progressively from 1.13 (BSH) to 0.97 (YCM) as the YCM content increases, indicating improved structural order and reduced defect density. This trend is attributed to the higher PAH content in YCM; during carbonization, strong π–π interactions between PAH molecules promote the extension of sp^2^-hybridized carbon networks and the formation of better-organized graphitic nanodomains, thereby progressively diminishing structural defects.

The N_2_ adsorption–desorption isotherms of BYCM-20, BYCM-40, and BYCM-60 are all type I, indicating a predominantly microporous nature (Figure 4d,e). According to Table S2, the specific surface area (SSA) increases from 1987.63 to 3098.11 m^2^·g^−1^ with increasing BSH proportion. This trend arises because the abundant alkyl side chains and oxygen-containing functional groups in BSH promote KOH activation [31], whereas the π–π stacking of PAHs in YCM densifies the carbon skeleton, restricting activator diffusion and hindering pore development [32]. The pore size distribution reveals that BYCM-40 possesses the most abundant pore size of 0.91 nm in the sub −1 nm range and a hierarchically organized porous structure (75% micropores and 25% mesopores). The oxygen-containing groups from BSH ensure micropore formation, while the moderate π–π stacking from YCM suppresses excessive micropore collapse and preserves mesopores. This suitable blending ratio (e.g., BYCM-40) yields a large SSA together with a hierarchically arranged combination of micropores and mesopores, helping transfer ions of different sizes.

XPS spectra confirmed the presence of C, N, and O. Elemental analysis (Table 2) showed that BYCM-40 possessed a lower C content (70.41%) and a higher O content (22.77%) compared to BYCM-20 and BYCM-60, suggesting that an optimal coal blending ratio favors oxygen-enriched porous carbon. High-resolution C 1s spectra were deconvoluted into sp^2^ C, sp^3^ C, C–N/C–O, and C=O [33]. BYCM-20 exhibited the highest sp^3^ C content due to stronger activation and defect formation, whereas BYCM-60 had the highest sp^2^ C content, reflecting greater graphitic ordering; BYCM-40 displayed a balanced sp^3^/sp^2^ ratio. O 1s spectra resolved into C=O, C–O, and C–OH groups, which enhance electrode wettability and pseudocapacitance [34]. N 1s spectra revealed pyridinic N-oxide, pyridinic N (N-5), and pyrrolic/pyridonic N (N-6); N-5 and N-6 are electrochemically active in alkaline electrolytes, improving wettability and providing additional pseudocapacitance [35]. The incorporation of N and O heteroatoms into the sp^2^ carbon network induces charge redistribution, generating pseudocapacitance and boosting overall capacitance. Consequently, BYCM-40, with its moderate SSA, well-developed pore structure, balanced defects/graphitization, and elevated oxygen content, achieves enhanced electrochemical performance.

### 2.4. Electrochemical Properties

**Figure 5:**
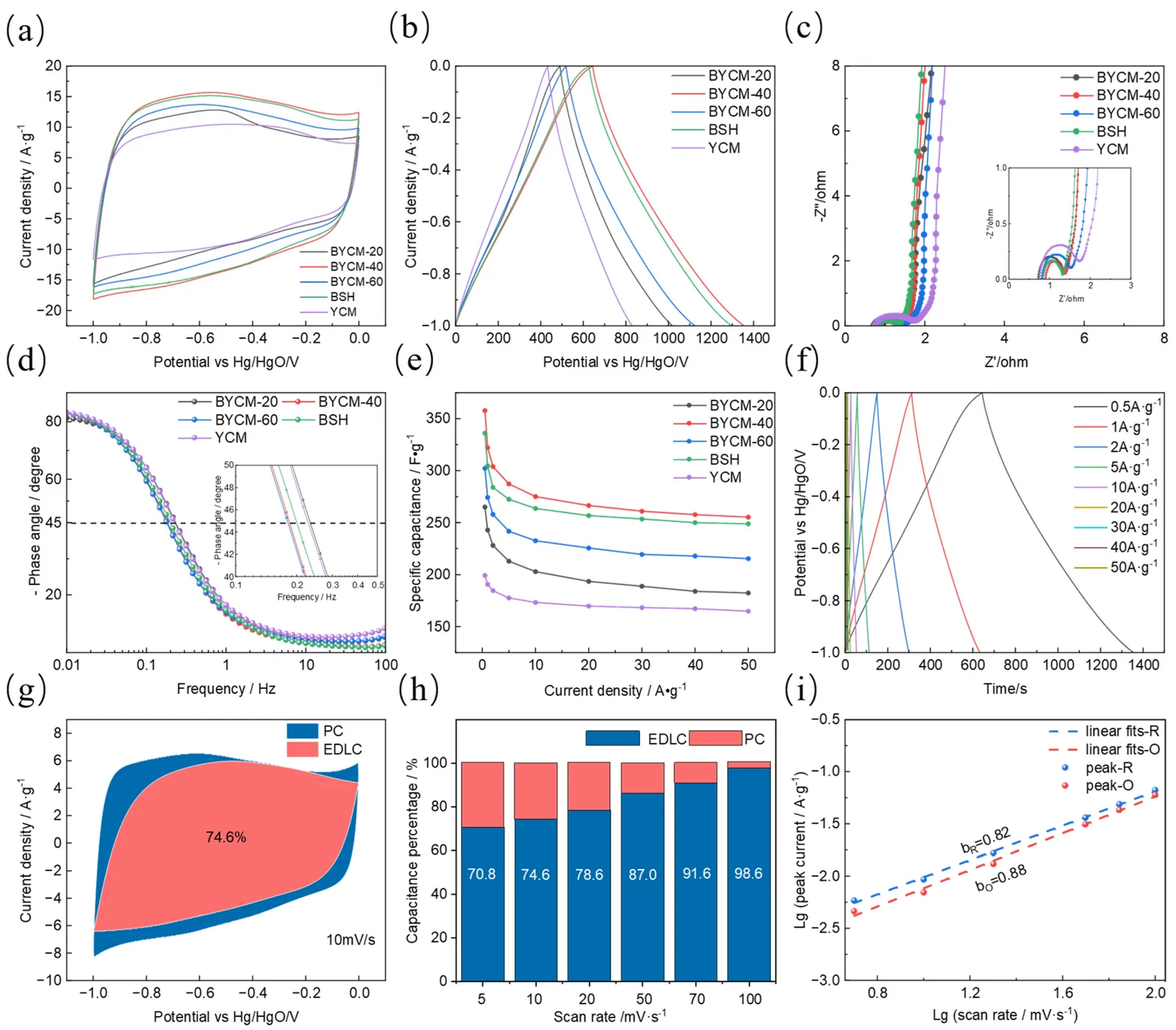
Electrochemical performance of BSH, BYCM-20, BYCM-40, BYCM-60, and YCM: (**a**) cyclic voltammetry (CV) curves at 50 mV·s^−1^; (**b**) Galvanostatic charge–discharge (GCD) curves at 0.5 A·g^−1^; (**c**) Nyquist plots; (**d**) Bode plots; (**e**) specific capacitance as a function of current density; (**f**) GCD curves of BYCM-40 at various current densities (0.5–50 A·g^−1^); (**g**) capacitive contribution of BYCM-40 at 10 mV·s^−1^; (**h**) capacitive contribution of BYCM-40 at different scan rates; and (**i**) log(i) vs. log(v) plot of BYCM-40.

Electrochemical measurements in a three-electrode configuration using 6 M KOH electrolyte revealed that all samples exhibit quasi-rectangular CV curves at 50 mV·s^−1^ (Figure 5a) and symmetric triangular GCD profiles at 0.5 A·g^−1^ (Figure 5b), confirming reversible capacitive behavior with fast charge/discharge response. Among them, BYCM-40 delivers the highest specific capacitance of 357.8 F·g^−1^ (Figure 5e), substantially outperforming BSH (336.1 F·g^−1^), BYCM-20 (265.1 F·g^−1^), BYCM-60 (302.2 F·g^−1^), and YCM (199.2 F·g^−1^). This superior capacitance is attributed to the synergistic combination of its high specific surface area (2556.53 m^2^·g^−1^), optimal mesopore proportion (25%), and elevated surface oxygen content (22.77 at%), which collectively promote efficient ion accumulation and enhanced charge storage.

EIS Nyquist plots (Figure 5c) show that BYCM-40 possesses the smallest high-frequency semicircle and the steepest low-frequency slope, corresponding to the lowest charge-transfer resistance (Rct = 0.47 Ω) and Warburg impedance (Zw = 0.77 Ω) [36]. The low Rct reflects facile interfacial charge transfer enabled by the well-developed porous structure, while the small Zw indicates rapid ion diffusion through the hierarchically interconnected pore channels. Its Bode phase angle of 85.6° (Figure 5d) approaches the ideal 90°, indicating a near-ideal capacitive response with fast frequency response. Kinetic analysis (Figure 5i) yielded b-values of 0.82 (cathodic) and 0.88 (anodic), confirming that capacitive-controlled processes dominate charge storage [37], with the EDLC contribution reaching 74.6% at 10 mV·s^−1^ (Figure 5g) and increasing further with scan rate (Figure 5h), consistent with the surface-controlled nature of the porous carbon electrode.

In a symmetric two-electrode configuration, BYCM-40 maintains a near-rectangular CV shape up to 50 mV·s^−1^ (Figure S3a), exhibits negligible ohmic drop in GCD curves across all tested current densities (Figure S3b), and retains 99.2% of its initial capacitance after 20,000 cycles with a Coulombic efficiency of 98.6% (Figure S3c), demonstrating outstanding rate capability and exceptional long-term cycling durability.

### 2.5. Quantitative Validation of the Synergistic Effect

To verify whether a genuine synergistic effect exists between low-rank Baishihu coal and high-rank Yicheng coal, rather than a simple additive effect, the experimentally measured properties of BYCM-40 were compared with the weighted predicted values based on the performance of the single-coal samples. The weighted predicted values were calculated using the following formula:

P_predicted_ = X_BSH_⋅P_BSH_ + (1 − X_BSH_)⋅P_YCM_

where X_BSH_ is the mass fraction of BSH in the blended coal (40% for BYCM-40), and P_BSH_ and P_YCM_ are the experimentally measured properties of the single BSH and YCM samples, respectively. The comparison results are summarized in Table 3.

The specific capacitance of BYCM-40 (357.8 F/g) was significantly higher than the weighted predicted value (approximately 254 F/g), representing an increase of 40.9%; the specific surface area, mesopore fraction, and oxygen content also exhibited positive deviations to varying degrees. These deviations clearly confirm that the mixed-coal strategy indeed produces a genuine synergistic effect that transcends a simple additive effect, rather than a linear superposition of the two coals’ properties. This synergistic effect can be attributed to the complementary roles of the two coals during the activation process: the abundant oxygen-containing functional groups in the low-rank coal provide a large number of active sites for the activation reaction, driving the full development of microporous and mesoporous structures; while the π-π stacking of polycyclic aromatic hydrocarbons in the high-rank coal effectively stabilizes the carbon framework, inhibiting pore structure collapse caused by excessive etching. The synergistic complementarity of these two coals in pore formation and structural stabilization enables BYCM-40 to outperform the simple weighted sum of the performance of either individual coal type in terms of capacitive performance.

## 3. Experimental Section

### 3.1. Materials

Baishihu coal originated from the Hami region of Xinjiang, whereas Yicheng coal came from Yili, Xinjiang. Prior to utilization, both coal samples were pulverized and sieved to a particle size of 200 mesh. The reagents potassium chloride (KCl), potassium hydroxide (KOH), polytetrafluoroethylene (PTFE), ethanol, and acetylene black were obtained commercially from Aladdin Reagent Co., Ltd., located in Shanghai, China.

### 3.2. Preparation of Electrodes and Testing of Electrochemical Properties

Coal-based porous carbon, acetylene black, and polytetrafluoroethylene (PTFE) were weighed in a mass ratio of 8:1:1. The materials were thoroughly mixed, and then the mixture was made into a slurry with ethanol. The slurry was evenly applied to a nickel mesh and compressed into a porous carbon working electrode using a tablet press. The electrode was dried at 80 °C for 12 h and set aside for later use. The loading of the active material was approximately 2 mg.

Electrochemical performance tests were conducted using a three-electrode system with 6 M KOH as the electrolyte, Hg/HgO as the reference electrode, a platinum sheet as the counter electrode, and a porous carbon electrode as the working electrode. Cyclic voltammetry (CV) tests (voltage window: −1 V to 0 V), charge–discharge curves (GCD), and electrochemical impedance spectroscopy (EIS) were performed. The frequency range for the AC impedance (EIS) test was from 0.01 Hz to 100 kHz.

Using 6 M KOH as the electrolyte (voltage window: 0 V to 1.2 V), a nickel mesh was cut into small circular disks with an area of 1 cm^2^. One milligram of active material was pressed between two nickel mesh disks to form an electrode sheet. Glass fiber was used as the separator, and a symmetric supercapacitor was assembled using a 2032-type button cell casing. The cells were encapsulated, and the long-cycle performance of the symmetric supercapacitors was tested using a Land battery testing system at 10 A·g^−1^. 

### 3.3. Synthesis of BYCM-X Coal-Based Porous Carbons

**Figure 6:**
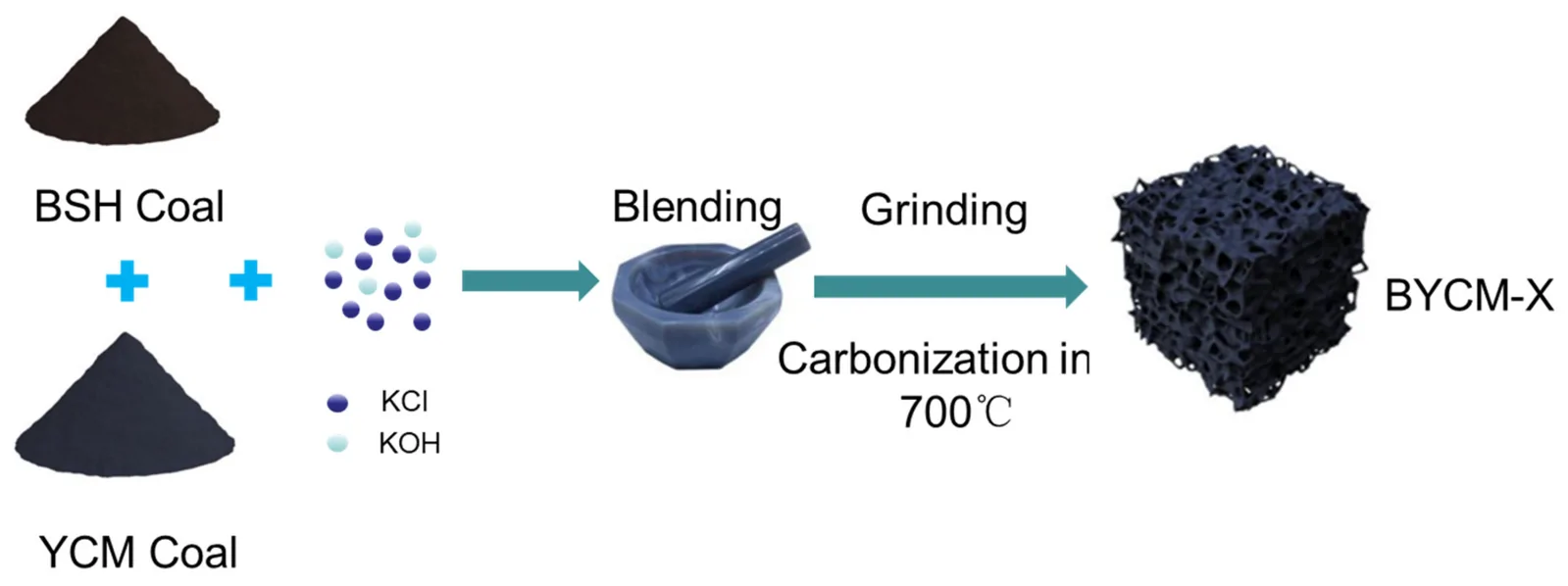
Schematic diagram of porous carbon preparation.

A mixture of Baishihu coal (0.2 g) and KOH/KCl (0.6 g KOH, 4 g KCl) was placed in a tube furnace, heated to 700 °C under a nitrogen flow at a ramp rate of 5 °C/min, and kept at this temperature for 1 h. Subsequently, the obtained powder was rinsed with 1 M HCl and then dried at 80 °C; the final product was labeled as BSH. The detailed steps of this preparation route are illustrated in Figure 6. Additionally, a mixture of coal (0.2 g, consisting of Baishihu coal and Yicheng coal) was carbonized with KOH/KCl (0.6 g KOH and 4 g KCl) under the same conditions; the resulting powder, after acid washing and drying, was designated as BYCM-X. The values X = 60, 40, and 20 represent the mass fractions of Baishihu coal. Furthermore, YCM was obtained by carbonizing pure Yicheng coal using the same BYCM-X preparation process.

### 3.4. Materials Characterization

X-ray diffraction (XRD) patterns were recorded on a Regulus8220 diffractometer (HITACHI, Chiyoda, Tokyo, Japan) using Cu Kα radiation (λ = 0.15406 nm) over a 2θ range of 10–80° with a step size of 0.02° to identify the crystalline phases and graphitization degree of the prepared carbon materials. The surface morphology of the samples was examined by field-emission scanning electron microscopy (FE-SEM, S-4800, Hitachi, Chiyoda, Tokyo, Japan) at an acceleration voltage of 5 kV. Thermogravimetric analysis (TGA) was performed on a STA449F3 instrument (Netzsch-Geratebau GmbH, Selb, Germany) under N_2_ atmosphere from room temperature to 800 °C at a heating rate of 10 °C·min^−1^ to evaluate the thermal stability and activation behavior of the raw coals and their mixtures with activators. Raman spectra were collected on an R200-L spectrometer (Bruker Optik GmbH, Ettlingen, Germany) with a 532 nm excitation laser to assess the structural disorder and graphitization degree of the carbon products. X-ray photoelectron spectroscopy (XPS) measurements were carried out on an ESCALab220i-XL spectrometer (VG Scientific, Fisons Instruments, West Sussex, UK) with monochromatic Al Kα radiation (hv = 1486.6 eV); all binding energies were calibrated using the C 1s peak at 284.8 eV. Specific surface areas and pore structures were determined by N_2_ adsorption–desorption isotherms measured at 77 K using a surface area analyzer (ASAP 2460, Micromeritics Instrument Corporation, Norcross, GA, USA), with the Brunauer–Emmett–Teller (BET) method for specific surface area and the Barrett–Joyner–Halenda (BJH) and non-local density functional theory (NLDFT) models for pore size distribution.

## 4. Conclusions

This study establishes a co-carbonization principle in which low-rank and high-rank coals play complementary yet competing roles: the abundant oxygen functionalities and alkyl chains in low-rank coal promote activation-induced pore generation, whereas the extensive π–π stacking of polycyclic aromatic domains in high-rank coal drives local graphitization but, in excess, restricts pore development through framework densification. An optimal blending ratio balances these antagonistic effects—decoupling micropore formation from structural ordering—and simultaneously tunes defect density. This synergy produces a hierarchically porous carbon with high specific capacitance and exceptional cycling stability, demonstrating that the rational blending of coals of different rank provides a straightforward, cost-effective route to high-performance supercapacitor electrodes. Building on this platform, future integration of deliberate heteroatom doping is expected to further elevate performance by introducing additional pseudocapacitance and favorably modulating the electronic structure, extending the design space for coal-derived carbon materials.

## Figures and Tables

**Table 1:** Char yield for BYCM-20, BYCM-40, and BYCM-60.

Sample	Char Yield (wt%)
BYCM-20	49.85
BYCM-40	39.70
BYCM-60	32.60

**Table 2:** Elemental composition of BYCM-20, BYCM-40, and BYCM-60.

Sample	XPS (at.%)			High Resolution of C 1s (%)			
	C	N	O	sp^2^C	sp^3^C	C-N/C-O	C=O
BYCM-20	85.31	0.94	11.75	28.9	48.5	12.2	5.9
BYCM-40	70.41	0.81	22.77	30.5	44.1	12.3	6.7
BYCM-60	80.26	1.06	14.88	41.1	36.8	11.3	5.6

**Table 3:** Comparison of specific capacitance of BYCM-40 with weighted predicted values.

Property	BSH	YCM	Predicted Value	BYCM-40	Enhancement
Specific capacitance (F/g)	336.1	199.2	~254	357.8	+40.9%

## Data Availability

Data is contained within the article.

## Supplementary Materials

The following supporting information can be downloaded at https://www.mdpi.com/article/10.3390/molecules31172997/s1, Figure S1: (a) XPS spectra of BYCM-20, BYCM-40, and BYCM-60 (b) C1s; Figure S2: Equivalent Circuit Diagram; Figure S3: (a) CV curves of the BYCM-40//BYCM-40 symmetric supercapacitor at different scan rates (10–50 mV·s^−1^) (b) GCD curves. Table S1: Industrial Analysis Table for Raw Coal; Table S2: Pore size information for the BYCM-20, BYCM-40, and BYCM-60; Table S3: Equivalent Circuit Parameters for the BYCM-20, BYCM-40, and BYCM-60; Table S4: Carbon, nitrogen, and oxygen content in raw coal.
