# Supplementary material for: Synergistic Co-Blending of Low- and High-Rank Coals: A Strategy for Engineering High-Performance Porous Carbons for Supercapacitors
Source: Molecules. 2026 Aug 27;31(17):2997. doi: 10.3390/molecules31172997 (PMC13567573; doi:10.3390/molecules31172997)
Supplement: Supplementary file 1 [file molecules-31-02997-s001.zip › molecules-4511962-supplementary.pdf]

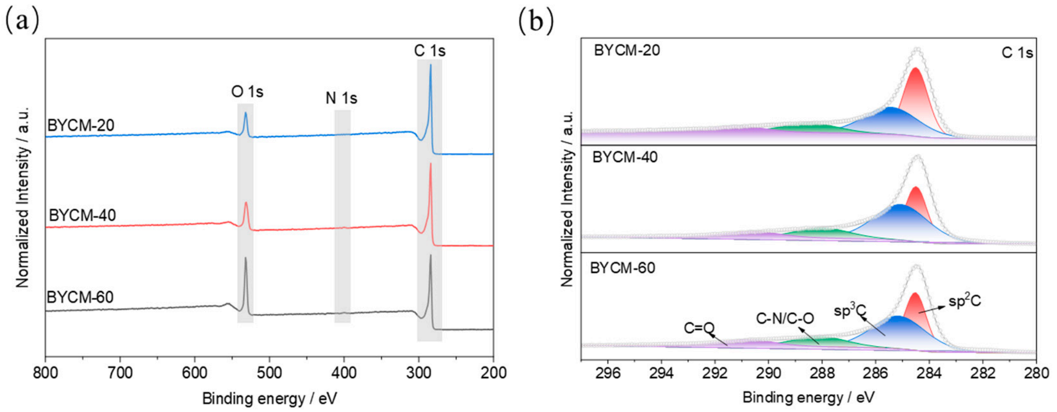

Figure S1 (a) XPS spectra of BYCM-20, BYCM-40, and BYCM-60 (b) C1s

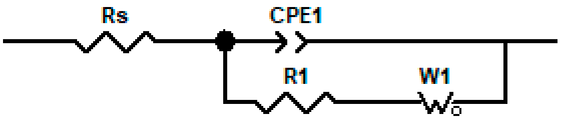

Figure S2 Equivalent Circuit Diagram

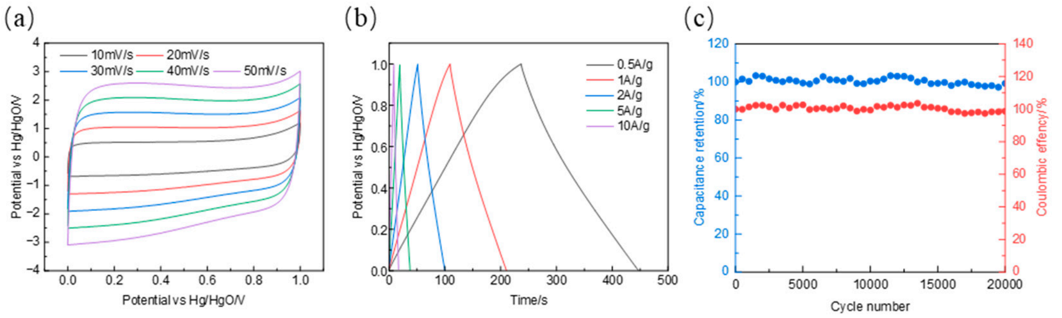

Figure S3 (a) CV curves of the BYCM-40//BYCM-40 symmetric supercapacitor at different scan rates (10–50 mV·s^{-1}); (b) GCD curves

Table S1 Industrial Analysis Table for Raw Coal

Sample	M_{ad}(%)	A_d（%）	V_{daf}(%)
BSH	18.07	4.66	46.34
YCM	0.70	6.37	23.10

Table. S2 Pore size information for the BYCM-20, BYCM-40, and BYCM-60

Sample	Surface area $m^2\ g^{-1}$	Total pore volume $cm^3 \cdot g^{-1}$	Micropore volume $cm^3 \cdot g^{-1}$	Mesopore volume $cm^3 \cdot g^{-1}$	Pore size nm
BYCM-20	1987	0.92	0.83	0.09	1.86
BYCM-40	2556	1.24	0.92	0.31	1.94
BYCM-60	3098	1.50	1.20	0.29	1.93

Table. S3 Equivalent Circuit Parameters for the BYCM-20, BYCM-40, and BYCM-60

Sample	Rs(Ω)	Rct(Ω)	Zw(Ω)	n	$Q(\times10^{-3}\Omega^{-1}s^{n})$	$\chi^2(\times10^{-3})$
BYCM-20	0.71	0.63	0.91	0.80	1.13	0.84
BYCM-40	0.89	0.47	0.77	0.88	0.91	0.75
BYCM-60	0.79	0.75	1.15	0.89	0.97	0.93

Table. S4 Carbon, nitrogen, and oxygen content in raw coal

Sample	XPS(at.%)			O-containing functional groups (%)		
	C	N	O	C-OH	C-O	C=O
BSH	74.51	2.09	19.90	29.71	17.13	53.16
YCM	76.79	1.49	16.52	34.16	14.48	51.36
